# Editorial

**Published:** 1864-09

**Authors:** 


### Editorial.

### THE AMERICAN DENTAL CONVENTION.

Met in Detroit on Tuesday the second of August. It was composed of about one hundred members of the profession from various parts of the country. We noticed several present who had never attended such a meeting before; and generally they were surprised and gratified to see the position in which the profession stood. All such had their interest awakened and their energies aroused; such will in the future work for the development and elevation of the profession. There were a few who were disappointed; they saw nothing and heard nothing interesting, or worthy their attention, one said he was much disappointed, for he did expect to learn something, but it had been a failure, and his valuable time and money were wasted. Had he been the only one in the world who could not learn anything, we should have felt different about the matter.

To nearly all who were present the meeting was an exceedingly profitable one. A new feature was introduced, viz.: that of clinic exercises, this called forth a great press for observation. It is proposed that hereafter such demonstrations shall enter more largely into the exercises of the meetings. Many people learn much more rapidly through their eyes than through their ears.

We trust the profession will be blessed with many such meetings in the future, and that a just appreciation may be entertained of them by every member of the profession, and such an appreciation as will lead them to attend all such meetings. We were unable to make any report of the discussions and proceedings, but will in the October number present our readers with the able report published in the *Dental Cosmos.* T.

### AMERICAN DENTAL ASSOCIATION.

This body met at Niagara Falls on the 28th of July ult.

All the Dental Societies in the United States, with one or two exceptions, were represented. There were over eighty delegates present,—earnest working men, who entered fully into the work before them. We never before witnessed such a meeting; there was the most earnest and deep searching investigation of subjects pertaining to the profession, and those too that have hitherto been largely over-looked. It is gratifying to see a growing disposition to study and deal with principles rather than special formulas.

The influence of this central organization will certainly permeate the entire profession and be productive of great good.

There is a great interest now manifested by the profession throughout the country in organizing and sustaining local societies. This interest is awakened to a great extent by this representative association.

We regard this organization as a permanent institution, we hope it may be equal in duration to the profession itself.

We shall not publish a report of its proceedings in the REGISTER, but refer our readers to the Transactions, which will be issued soon; a copy of which every Dentist should procure. T.

### TRANSACTIONS OF THE ODONTOGRAPHIC SOCIETY OF PENNSYLVANIA.

We have just received the volume of Transactions of this Society, and regard it as quite an acquisition to our library. It embraces a large amount of valuable and interesting matter, the result of the first year's labor of the society. The essays and discussions are of a high order, and will compare favorably with those of any professional or scientific body with which we are acquainted.

This volume should be in the hands of every member of the profession, and especially should all members of societies have in their possession all such works.

This volume of Transactions may be obtain of Professor J. H. McQuillin, Philadelphia, Pa., Corresponding Secretary of the society. T.

### PHYSICIANS' VISITING LIST, AND BOOK OF ENGAGEMENTS.

This little work prepared and published by Lindsay & Blakiston, of Philadelphia, for 1865, is upon our table. It is the most complete and perfect in its arrangement of anything we have seen of the kind. It contains a division for each department of medical practice. There is a full visiting list and memoranda pages for each month, pages for the address of patients and others—also for a list of bills and accounts, and for obstetric engagements and memoranda, vaccination, &c., &c. The work is very neatly gotten up,—one that no physician should be without. It is sold by Robert Clarke & Co. of this city Price $1.25. T.

### MEETING.

The Western New York Dental Association, holds its semi-annual meeting at Lockport, New York, on Tuesday the 4th of October next, when we presume there will be a full gathering of the profession of Western New York.

The profession is fully awakened to the importance of associative effort. Already the meetings of this society have done much good ; and if they are well sustained we know still greater good will result.

We would suggest that all within reach, should be in attendance and participate in the work. No one knows the extent of his loss, who refuses the aid proffered to him by his brethren. Usually those most deficient are the slowest to avail themseves of the advantages of association.

We expect a good report from the Western New York Society.
T.

### NEW TEETH.

We have recently used some vulcanite teeth, manufactured by Rubencame & Stockton, without pins, the attachments being effected by means of mortices and dovetails. The attachment in those we have used is very good, and in the great majority of cases can be used. Where it is necessary to use very short teeth, or where much grinding is required they may be objectionable, but even pins may be ground out as well as mortices and dovetails. But it seems where a small tooth is required this method of attachment will render the tooth frail. We grant our liability, however, to be mistaken in this opinion. But all cases where a considerable amount of tooth body can be used, these teeth will answer well. T.

### OHIO COLLEGE OF DENTAL SURGEONS.

The regular course of lectures will commence in this institution on the first Monday of November next, under very encouraging auspices. Arrangements have been made which relieve the College entirely from the pecuniary embarrassment that so long rested upon it, and cramped its operations. The vacancy occasioned by the resignation of Prof. H. R. Smith, of the chair of Mechanical Dentistry, has been filled by the appointment of Dr. J. Cheesbrough of Toledo, Ohio, one whom we regard as eminently qualified for the position. Large additions will be made to each department of the College, and especially will the laboratory and infirmary be fitted up in the best possible manner. The Clinics and demonstrations will be under, the immediate supervision and direction of the Professors of Operative and Mechanical Dentistry.

The present indications are that there will be a good class.

T.

### OHIO MEDICAL COLLEGE.

The annual announcement of this Institution is upon our table.

There has recently been several changes in the faculty, though we are assured they are such as will not lessen the efficiency of the course. This is a veteran institition and stands in the front amongst the Medical Colleges in the West, and now as it ever has done commands the high regard of the profession of the whole country. T.

